# Stochastic assessment of management strategies for a Mediterranean peri-urban wild boar population

**DOI:** 10.1371/journal.pone.0202289

**Published:** 2018-08-29

**Authors:** Carlos González-Crespo, Emmanuel Serrano, Seán Cahill, Raquel Castillo-Contreras, Lluís Cabañeros, José María López-Martín, Joan Roldán, Santiago Lavín, Jorge Ramón López-Olvera

**Affiliations:** 1 Wildlife Ecology & Health Group and Servei d’ Ecopatologia de Fauna Salvatge (SEFaS), Departament de Medicina i CirurgiaAnimals, Facultat de Veterinària, Universitat Autònoma de Barcelona (UAB), Bellaterra, Barcelona, Spain; 2 Departamento de Biologia & Cesam, Universidad de Aveiro (UA), Aveiro, Portugal; 3 Consorci del Parc Natural de la Serra de Collserola, Barcelona, Spain; 4 Departament d’Agricultura, Ramaderia, Pesca i Alimentació, Serveis Territorials de Barcelona, Generalitat de Catalunya, Barcelona, Spain; 5 Forestal Catalana SA, Generalitat de Catalunya, Barcelona, Spain; University of Tasmania, AUSTRALIA

## Abstract

Wild boar (*Sus scrofa*) population spread into urban and periurban areas has exacerbated conflicts with humans. There is a need for planned wild boar management strategies, and Population viability analysis (PVA) combined with perturbation analyses allow the assessment of the management effort of control methods. Our study aims to develop stochastic predictive models of the increasing wild boar population of the 80 km^2^ peri-urban Mediterranean area of Collserola Natural Park (CNP), located near Barcelona, Spain, as well as assessing specific management measures (including reduced food availability, selective harvest, and reduction in fertility). Population parameters were estimated from previously published census and hunting data provided by the CNP and the local hunting administration. The results revealed that under the current conditions the CNP wild boar population will continue to increase. The most efficient strategy to reduce wild boar abundance was a combination of reducing supplementary anthropogenic food resources and selective removal of juvenile (<1 year) and yearling (1–2 years) wild boar. These strategies will probably be also the most efficient ones in other oversupplemented increasing wild boar populations in similar situations, although specific studies will be needed to fine-tune the best management option for each context. PVA allows the prediction of future population trends and the assessment of the efficacy and efficiency of potential management strategies before implementing management measures.

## Introduction

Wild boar (*Sus scrofa*) population numbers have increased and their distribution area has spread worldwide in the last decades, mainly due to artificial feeding, a reduction in predators and translocations [1], changes in land use and decrease of human population in rural areas [2–4]. At least in Europe, climate change is also favoring wild boar populations through milder winters and increased mast productivity [5]. As a generalist species, the wild boar is capable of successfully colonizing and exploiting a wide range of habitats [6], including the interface between urban areas, agricultural landscapes and even highly artificial urban green areas [7,8].

Increasing wild boar population in rural areas and in proximity to urban areas has exacerbated conflicts with humans. Wild boar cause damage to crop fields in cultivated areas, to plant diversity, vegetation composition and regeneration patterns [9,10], they prey on a number of animal species like ground-nesting birds, such as red-legged partridge (*Alectoris rufa*), pheasants (*Phasianus colchicus*), mammals as the red-backed vole (*Clethrionomys gapperi*) and short-tailed shrew (*Blarina brevicauda*) and even domestic livestock[11]. Wild boar are increasingly involved in vehicle collisions [7,8,12,13]. The colonization of urban areas and habituation to humans has also increase damage in parks, green areas, attacks on people and pets, and pose human-health risks [7,8,14].

Regulated wild boar hunting has been the primary method of population control. However, wild boar hunting is declining in some European countries and is currently insufficient to halt wild boar population growth [4]. Suggested methods to control the growth of wild boar populations include the use of toxicants, not approved in Europe but common in other parts of the world such as Australia [15] and fertility control [10,16]. Other methods are aimed to decrease damage and conflicts like the use of repellents, translocation and fencing [10,17]. However, none of these methods provides a definitive solution to control population growth because the high reproductive rate of wild boar compensates for the potential mitigation effects of these measures [17,18].

There is a general need for carefully planned wild boar management strategies [9]. Identifying the vulnerable life stages of pest species and their relative responses to perturbations [19,20] allows the establishment of control methods within the proper focus for management effort [21]. Population viability analysis (PVA) combined with perturbation analyses (i.e. sensitivity and elasticity) are currently the most commonly used methods for this objective [20].

Population Viability Analysis (PVA) is a model-based quantitative risk assessment that, relying on ecological models, identifies the viability requirements and threats to a species population, also evaluating the likelihood of persistence, either for a given time under current conditions or expected from proposed management. Although PVAs were originally developed for threatened species to evaluate the risk of extinction allowing to minimize the risks [22,23], they have also been used to evaluate the impact of disease outbreaks [24] and to assess the effects of management measures aimed at reducing population size for invasive and pest species [20,23].

Both PVA and sensitivity analyses can also be used as a decision-support tool to identify key life cycle stages and/or demographic processes as targets for management interventions for established invasive species [20,23]. This allows the determination of the most cost-efficient management strategies [25] and the effect of different management strategies prior to undertaking them.

The purpose of our study was to develop stochastic predictive models of the wild boar population of the peri-urban Mediterranean area located near Barcelona, Spain. We specifically wanted to use sensitivity analyses [23] to identify the life stages (sex and age) to be targeted with specific management measures (including reduced food availability, selective harvest, and reduction in fertility), in order to achieve the maximum effect for population reduction [26]; and, secondly, to evaluate the effectiveness of the aforementioned management strategies on affecting the most vulnerable life stages and thereby controlling population growth. The results will provide managers with measures that can be applied to reduce wild boar populations and the attractiveness of urban areas for this species in Mediterranean ecosystems.

## Methods

**Fig 1:**
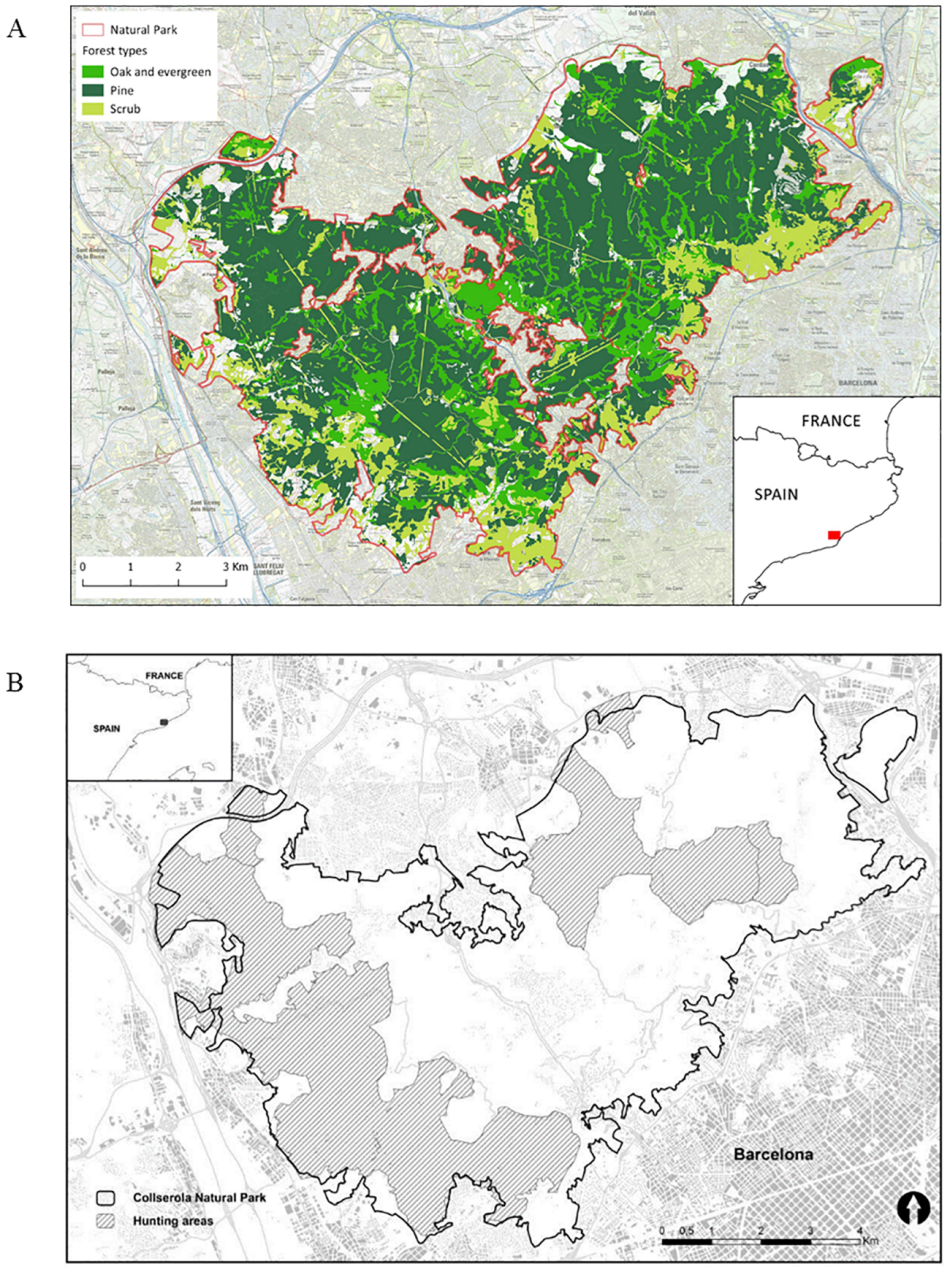
Study area. Maps of Collserola Natural Park, Barcelona, NE Spain, showing a) the different habitats and b) Controlled Game Area, currently the only hunting areas in the whole massif.

Our study area consisted of the 80 km^2^ Natura 2000 Collserola Natural Park (CNP) (41°25'52''N, 2°4'45''E), located in Barcelona, in north-eastern Spain, Wild boar are considered abundant in the province of Barcelona [14,27]. The CNP (Fig 1) is surrounded by urban areas within the Barcelona metropolitan area (AMB, by its acronym in Spanish). The AMB is one of the largest European metropolitan areas, with 36 municipalities occupying more than 636 km^2^ and populated by 3.2 million people (population density of 5,000 people per km^2^) (AMB 2015).

The CNP is virtually isolated from the nearby natural and agricultural areas by urban development and road and train networks (Fig 1A) [14], although some corridors and ecological connectors, such as riparian areas and dry riverbeds, are used by wild boar, hence allowing some movements out of this area [28]. The CNP is a Mediterranean hilly area, with an altitude ranging from 60 to 512 meters at the Tibidabo summit. The climate is typically Mediterranean, with warm dry summers and mild wet winters. Annual rainfall is 672 mm and average annual temperatures range from -4°C (minimum) to 35°C (maximum). The vegetation of CNP is mainly composed of Mediterranean scrub (24%) and mixed woodland of Aleppo pine (*Pinus halepensis*) (40%) combined with evergreen oak (*Quercus ilex*) (15%) and deciduous oak (*Q*. *cerrioides*) (0.7%) [7,29]. The remaining surface is composed by abandoned fields, wastelands and ruderal areas (3.9%), urban areas (3.5%), herbaceous (2.1%) and woody (1.8%) croplands and others (i.e. grasslands, ports, rafts, artificial canals, etc.) (9.0%). Oak acorn production in Mediterranean areas is highly variable, both intra- and inter-annually, mainly due to spring weather conditions during flowering and acorn growth [30,31]. Inter-annual evergreen oak production variation in Catalonia ranges from 58 to 82% [32], and a full mast year takes place every four years on average [32,33]. The wild boar is the only wild ungulate and the largest animal in size inhabiting the CNP. Although some minor piglet predation by medium-sized carnivores such as foxes may happen within the park, no natural predators for adult wild boar thrive inside the park. Therefore, natural predation is likely negligible and has no impact on the wild boar population dynamics.

The wild boar population in the CNP has increased and become habituated to human presence, due to anthropogenic resources, including street bins, waste containers, stray cat colonies, urban green areas and direct feeding by people (Figures A and B in S1 File) [28]. Anthropogenic feeding is facilitated by the proximity of densely vegetated areas close to the city limits [7,34,35].

In CNP, hunting is allowed from October through February as a traditional activity with a management plan in the Controlled Game Area of Collserola, which comprises 38% of the CNP surface and the same proportion of habitats described for the CNP (Fig 1b). Hunting is carried out via drive hunts with hunters at fix positions and hound packs flushing the boars, in about 17.2% of the park. In an attempt to reduce wild boar abundance and damage, hunting pressure has progressively increased since 2004 through night waits (single hunter from a fix position, using bait and spotlights but not hide), granted almost year-round even in non-hunting areas after damage claims[7].

In spite of such hunting pressure, the estimated CNP wild boar population has experienced a 10-fold increase from 2000 to 2015, reaching an estimated relative abundance of around 1,500 wild boar (Table 1). The estimated percentage of harvested wild boar with respect to the estimated wild boar population increased throughout the study period from 10.0% (2000–2003) to 46.5% (2012–2015) (Table 1). However, adults were overrepresented (65.6%) in the battue hunting bag as compared to their proportion in Mediterranean populations (25%) [1,18,36,37], whereas yearlings and juveniles accounted only for 34.4% of the total harvest, far less than their proportion (75%) [1]. Although the scarce detected poaching have been included in the mortality rate, both the amount of wild boar poached and the effect of poaching on the CNP wild boar population are negligible.

**Table 1:** Wild boar harvested and abundances in Collserola Natural Park from 2000 to 2014.

Year	Hunting season	Estimated wild boar population in CNP (CI 95%)	Wild boars hunted in drive hunts	Wild boars hunted in night waits	Registered mortality (% of the estimated population)^+^
2000	2000/2001	165 (0.0–371.4)	19	0	19 (11.5)
2001	2001/2002	357 (167.8–546.2)	35	0	35 (9.8)
2002	2002/2003	191 (15.4–366.6)	18	0	18 (9.4)
2003	2003/2004	280 (98.0–462.0)	27	0	27 (9.6)
2004	2004/2005	579 (400.2–757.8)	61	19	128 (22.1)
2005	2005/2006	-	26	35	129
2006	2006/2007	558 (295.7–820.3)	26	43	136 (24.4)
2007	2007/2008	689 (485.5–892.5)	77	37	173 (25.1)
2008	2008/2009	-	29	44	171
2009	2009/2010	809 (580.1–1,037.9)	50	53	168 (20.8)
2010	2010/2011	821 (608.0–1,034.0)	72	77	222 (27.0)
2011	2011/2012	773 (458.5–1,087.5)	84	108	269 (34.8)
2012	2012/2013	1,050 (786.1–1,313.9)	109	171	462 (44.0)
2013	2013/2014	759 (596.1–921.9)	114	261	486 (64.0)
2014	2014/2015	831 (662.6–999.4)	75	206	326 (39.2)
2015	2015/2016	1,500 (1,296.5–1,703.5)	123	432	650 (43.3)

^+^ Including all the wild boar hunted, killed in car accidents, poached and captured and euthanized.

Fertility control of the wild boar population of the CNP has not been attempted, and repellents are unlikely to be effective in reducing the impact of wild boar [10]. Finally, fencing of CNP is incompatible with the human uses of this natural area surrounded by a 3.2 million human population.

All the data have been gathered from hunting records and wild boar management projects but no wild boar has been hunted, captured, handled or euthanized for this study.

### Data collection

Sex, age and abundance data for the local wild boar population (Tables 1 and 2) were collected by the authors from wild boar captured, hunted or found dead from 2000 to 2015. According to age-specific variation in demographic parameters we defined three age classes for each sex [36,38,39]: juveniles (0–1 years), yearlings (1–2 years) and adults (> 2 years). Specific age class abundances were calculated from the aforementioned data collected by the authors and were used to calculate the specific age class mortality rates (Table 2).

**Table 2:** Input data used in the model scenarios of the Collserola Natural Park wild boar population. Life history and population attributes: A) Reproduction values; B) Mortality and environment values. EV: Environmental variation.

**A**				
**Parameters**			**Base value**	**Source**
**Breeding system**			Polygynous	[12]
**Age of first offspring (year)**		Females	1	[12,27]
		Males	2	
**Maximum age of reproduction (year)**		Female	11	[12]
		Male	11	
**Maximum lifespan (years)**			11	[12]
**Maximum of broods per year**			2	[39,40]
**Maximum of progeny per brood**			6	[12]
**Sex-ratio at birth**			1:1	[27]
**% females breeding** **(SD due to EV)**		0–1 years	15 (10)	[12,27]
		1–2 years	60 (10)	
		> 2 years	70 (10)	
**Distribution of broods per year**		0 broods	10	[39,40]
		1 brood	85	
		2 broods	5	
**Number of offsprings**		Mean (SD)	3.5 (2)	[27]
**% males in the breeding pool**			25	[27]
**B**				
**Parameters**			**Base value**	**Source**
**Mortality rates**^+^ Mean as % (SD due to EV)	Females	0–1 years	29 (10)	Present study
		1–2 years	35 (10)	
		> 2 years	39 (10)	
	Males	0–1 years	30 (10)	
		1–2 years	43 (10)	
		> 2 years	35 (10)	
**Catastrophes**				
**1) Severe drought**				
Frequency			15%	Servei Meteorologic de Catalunya, unpublished data
Severity	Reproduction^a^		0.5	[27,41,42]
	Survival^b^		0.5	[9,36,43]
**2) Full mast**				
Frequency			22%	[32,33]
Severity	Reproduction^a^		1.5	[27]
	Survival^b^		1.5	[9]
**Carrying capacity (K)**				
K value (SD due to EV)			3000 (150)	Present study

^+^We estimated the age-class survival rates (*Sac*) from hunting data using the formula [44,45]: $Sac=\frac{\sum Nac+1(tx+1)}{\sum Nac(tx)}$, where *Nac* and *Nac* + 1 are the abundances of the ageclasses, and *tx* the census years. *Nac* were calculated from data collected by the authors.

^a^ Proportion of wild boar reproducing.

^b^ Proportion of wild boar surviving.

Population trend was estimated from hunting bags only from the drive hunt data (S2 Table), a reliable index of wild boar relative abundance [37]. Briefly, the number of wild boar hunted in every hunting event is divided by the hunted surface. This value is corrected by the mean efficiency of the hunting season (total wild boar hunted divided by the total wild boar seen in all the drive hunts of the year) and the result is again divided by the ratio between the number of hunting events in a season and the mean annual number of hunting events. This method was used consistently during the whole study period with minor variations among years (except for 2005 and 2008) in the independent variables: number of drive hunts (18.4 SD 0.97), number of hunting days (9.2 SD 0.48), hunters in each drive hunt (44.1 SD 3.43) and dogs in each drive hunt (46.5 SD 2.96).

We obtained reproductive data (Table 2) from literature review on wild boar biological parameters in neighboring populations in Mediterranean environments [1,18,27,46].

To provide a carrying capacity (K value) allowing to perform our PVA with VORTEX [48], we defined a hypothetical population threshold (HPT) fixed to a number of 3,000 individuals. This value falls just between the 1,000 wild boar value corresponding to a density of 12.5 wild boar/km^2^in the 80 km^2^ CNP [12,49], and the 6,400 wild boar corresponding to the maximum wild boar population density value recorded in fenced, food, water and shelter-supplemented Mediterranean environments (80 wild boar/km^2^, Gonçalves-Blanco, Ingulados Co., personal communication).

To include environmental stochasticity in the model, we considered the plasticity of Mediterranean wild boar populations, modeling a population characterized by high reproductive rates and high mortality in the first year of life [1], intense responses to food availability and weather conditions, with the proportion of reproducing females varying from 20–30% to 90% depending on food resource availability [39,46,49]. Altogether allows the population to increase even under yearly hunting pressures over 50% [38].

### Modeling

The trend of the CNP wild boar population was modeled from the published and estimated data (Table 2) over a time frame of 36 years (2000–2035) through two scenarios: past and future. We carried out simulation models using VORTEX Version 10.0.8 [47], a free software developed by the Chicago Zoological Society. The software is an individual-based simulation model for PVA that modeled the effect of deterministic and stochastic processes on the dynamics of wildlife populations [45].

We ran 500 iterations for each scenario to allow standard error calculations and we delayed the first year mortality until all annual mortality was done [45], in order to allow the harvest of juveniles. We included the vortex option of “environmental variation concordance of reproduction and survival” as the environmental variation affect reproduction and survival simultaneously [39,43] but not inbreeding effects, nor genetic management or density dependence effects on reproduction in the model.

*Past scenario*-We ran a 16-year (2000 to 2015) simulation with an initial population size of 165 wild boar (the estimated population size in 2000, CNP) to validate the model. We used the HPT value (3,000 individuals) and the parameter values introduced in VORTEX (Table 1). The number of wild boar of each age class harvested each year was modeled through a function (S1 Table).

*Future scenario*—A 20-year projection was run to study the future evolution of the population and to test both the impact of the variation in demographic rates and management strategies on the CNP wild boar population trend. The values for the parameters were taken from the past scenario, initiating the model with a wild boar population size of 1,500 individuals in 2015 (as estimated by the hunting bag analyses and confirmed by the past scenario model). Harvest was calculated to remain at 30% of the population, maintaining the same harvest proportion of each age-class as in the past scenario, and it was modeled by a function (S1 Table). We evaluated three HPT values (3,000, 4,200 and 6,400), corresponding to three different situations depending on the availability of anthropogenic resources under the same management.

### Sensitivity and elasticity analyses

Sensitivity and elasticity analyses estimate respectively the impact of absolute and proportional changes in biological parameters on population growth rate [21]. We tested the sensitivity and elasticity of the CNP wild boar population parameters on wild boar population trend in the CNP for 25 years in the future scenario, using the Sensitivity test (ST) implemented in VORTEX 10. We measured the sensitivity or impact as the total variation in the projected population sizes between the minimum and maximum value of the variable, and the elasticity or effect as the average population variation corresponding to each 10% parameter variation. The demographic variables were modified as follows to estimate the effect of three main different management strategies: 1) decreasing CNP HPT for wild boar (minimum value 500, maximum value 6,500, increment by 500) corresponding to different levels of anthropogenic food availability [9,10,17,50]; 2) reducing the percentage of breeding males and females (minimum value 0, maximum value 100, increment by 10) in each age-class through fertility control corresponding to variable fertility control effort [10,39]; and 3) increasing mortality (minimum value 0, maximum value 100, increment by 10) in sex and age-classes and a combination of them corresponding to variable and selective harvest pressure [1,17,18,38,50]. We also ran a factorial sensitivity analysis on the harvest values to estimate the best combination.

### Evaluation of management strategies

Once the ST determined the sensitivity and elasticity of the demographic parameters of the CNP wild boar population, we tested the effectiveness of reducing supplementary food availability and increasing selective harvest on modifying the variables selected by the sensitivity analyses in the future scenario. We did not evaluate the effectiveness of fertility control because the ST results of this strategy revealed a low effect on the variation in the projected population size. The output of each strategy was measured as the probability (PVA parameter “extinction probability”) of reaching the target population value and as the resulting wild boar population, both at the end of the future scenario period (25 years). The target population size (“extinction”) was set at 500 wild boar, half the theoretical natural carrying capacity of CNP (1,000 wild boar [12]), since this 50% value maximizes recruitment [51]. Wild boar is a native species in the CNP and the aim is not eradicating this species from the CNP but maintaining the population below ‘threshold’ levels not causing negative impacts in the ecosystem [11].

We modeled the decrease of CNP HPT for wild boar through supplementary feeding reduction [9,10,17,50] from the current estimated HPT value of 3,000 to the target value of 1,500, assuming a minimum supplementary food availability for 500 wild boar over the environmental carrying capacity (1,000 wild boar, [12]). We modeled such a decrease at two different rates: an idealistic option, with a 15% annual decrease for 5 years, and a conservative option, with a 5% annual decrease for 15 years. Secondly, we modeled the effectiveness of selective harvest [1,17,18,38,50], focused on increasing harvest in the best combination of values for juveniles and yearlings of both sexes provided by the sensitivity test results. Finally, we also modeled the effectiveness of an integrated management plan including the combination of supplementary feeding reduction and selective harvest.

## Results

### The past scenario

The population model calculated a population of 1,560 (34.42 SE) wild boar in 2015, agreeing with the evolution of the CNP wild boar population estimated from hunting bags, from 165 wild boar in 2000 to 1,500 in 2015. The deterministic annual increase (r) in wild boar abundance calculated by VORTEX was 0.3723.

### The future scenario

**Fig 2:**
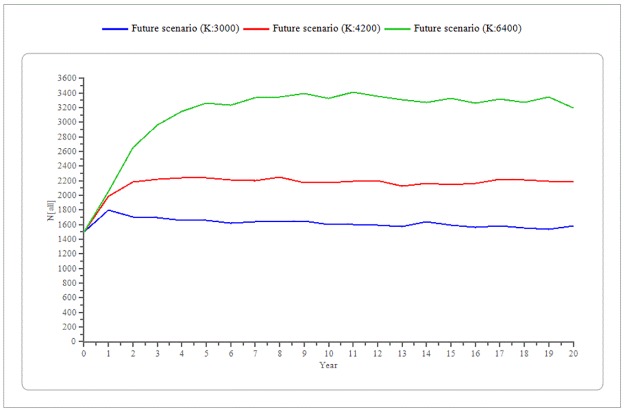
Predicted wild boar population trends. Future scenario results showed a progressive increase in the Collserola Natural Park final population size of: 1,673 wild boar for a K value (Hypothetical population threshold: Anthropogenic food resources availability) of 3,000, 2,281 wild boar for a K value of 4,200 and up to 3,304 wild boar for a K value of 6,400. Lines indicate SE.

The VORTEX simulations predicted that under the current conditions the CNP wild boar population will increase an 11.5% (until 1,673individuals, 26.81 SE), with an 8% probability of decreasing below the target size (500 individuals). Increasing HPT (K value) to 6,400 produced a consequent progressive increase in the final population size up to a 120.3% (until 3,304 wild boar, 61.56 SE) while the probability of achieving the target population size decreased to 4% (Fig 2).

### Sensitivity analyses

**Fig 3:**
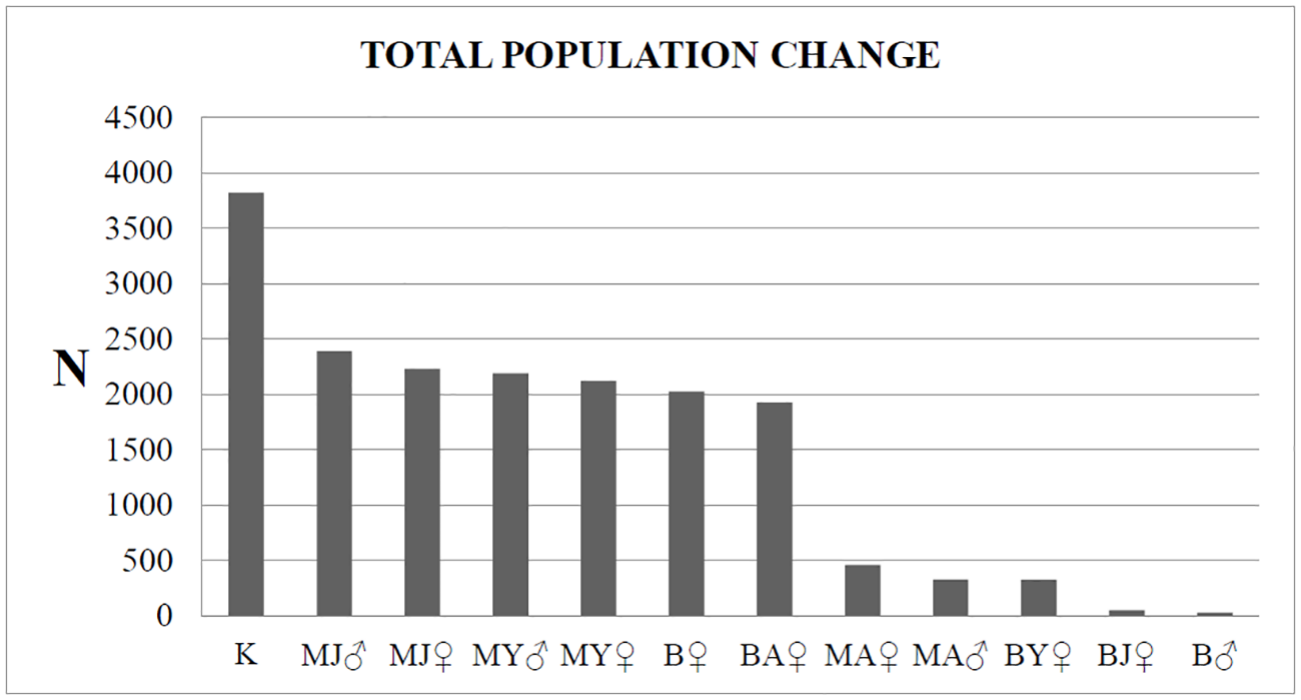
Impact of different parameters on wild boar population size according to the sensitivity tests. Total decrease (impact) in the wild boar population size of the Collserola Natural Park, Spain, for each parameter tested. K, Hypothetical population threshold: Anthropogenic food resources availability; M, Mortality; B, Breeding; J, Juveniles; Y, Yearling; A, Adults.

**Fig 4:**
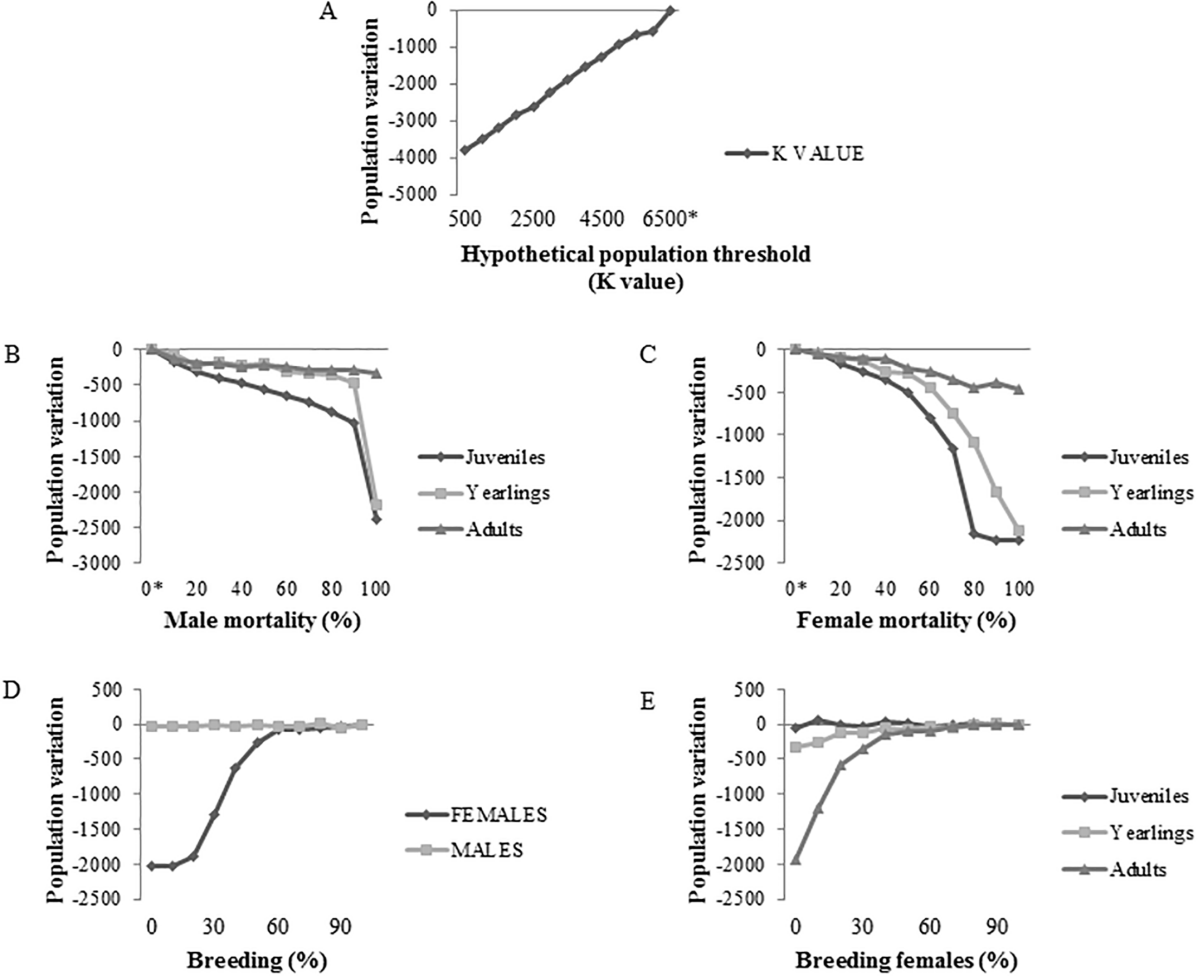
Effect of different parameters on wild boar population size according to the sensitivity tests. Wild boar population size of Collserola Natural Park, Spain, decrease for every 10% change of each of the parameters tested (A, Hypothetical population threshold: Anthropogenic food resources availability K value (Hypothetical population threshold: Anthropogenic food resources availability); B, male mortality; C, female mortality; D, breeding; E, breeding females). Breeding is the mean percentage of wild boar that breed in a given year (*, reference value).

The sensitivity analyses evidenced (Table 3, Figs 3 and 4) food availability as represented by HPT as the most influential parameter in population size (S2 Fig), followed by the mortality rate of juvenile males and females, and the mortality rate of yearling males and females. The impact of adult male and female mortality on the variation in the CNP wild boar population size was not significant (S3 Fig). Overall, the variations in female mortality rate had a stronger effect on population size than male mortality rate for all age-classes (Fig 4).

**Table 3:** Sensitivity test results for the different parameters tested.

Parameter tested in the sensitivity test (minimum-maximum value)			Relative population variation^a^		Variation between values (number of individuals)
			At minimum value	At maximum value	
**Hypothetical Population Threshold** **(500–6500)**			-79.6%	+175.0%	3,820
**Mortality** **(0–100%)**	**Juvenile**	**Male**	-100.0%	+59.1%	2,387
		**Female**	-99.4%	+49.0%	2,226
	**Yearling**	**Male**	-100.0%	+47.9%	2,187
		**Female**	-98.5%	+42.5%	2,116
	**Adult**	**Male**	+25.0%	+46.7%	325
		**Female**	+14.1%	+44.6%	457
**Breeding** **(0–100%)**	**Males**		+27.5%	+29.4%	28
	**Females**	**All age-classes**	-100.0%	+34.9%	2,024
		**Juvenile**	+25.7%	+29.0%	49
		**Yearling**	+13.5%	+34.9%	320
		**Adult**	-93.7%	+34.7%	1,925

^a^ The sign indicates the direction of the variation (+, increase; -, decrease)

Regarding reproduction, the variation in the percentage of reproductive females had a stronger impact on population size than for males (Table 3). Among females, the impact on wild boar population of the percentage of reproducing females increased with age. However, even though the variation in the predicted CNP wild boar population size due to the variation in the percentage of breeding females was high, only percentages of adult breeding females below 30% had an effect in achieving a significant reduction in CNP wild boar population size (Fig 4, S4 Fig).

The sensitivity analyses showed that a mortality rate between 40–60% for both juvenile and yearling wild boar, combined with a reduction of CNP HPT to a value of 1,500 wild boar, were the most effective measures to control and reduce the CNP wild boar population. Therefore, HPT and juvenile and yearling mortality were the variables selected by the model and consequently defined as target values for the management strategies (Table 4).

**Table 4:** Evaluation of the management strategies assessed in the model.

Management strategy	Effectiveness (success probability)	Years to reach target population size	Remaining abundance (N)
**Decrease supplementary feeding**	54^a^-56^b^ %	6^a^-15^b^	626^a^-636^b^
**Selective harvest**	70%	20	1651
**Combined**	100%	5^a^-10^b^	<501

^a^ Idealistic option (annual decrease of 15% during 5 years);

^b^ Realistic option (annual decrease of 5% during 15 years).

### Evaluation of management strategies

**Fig 5:**
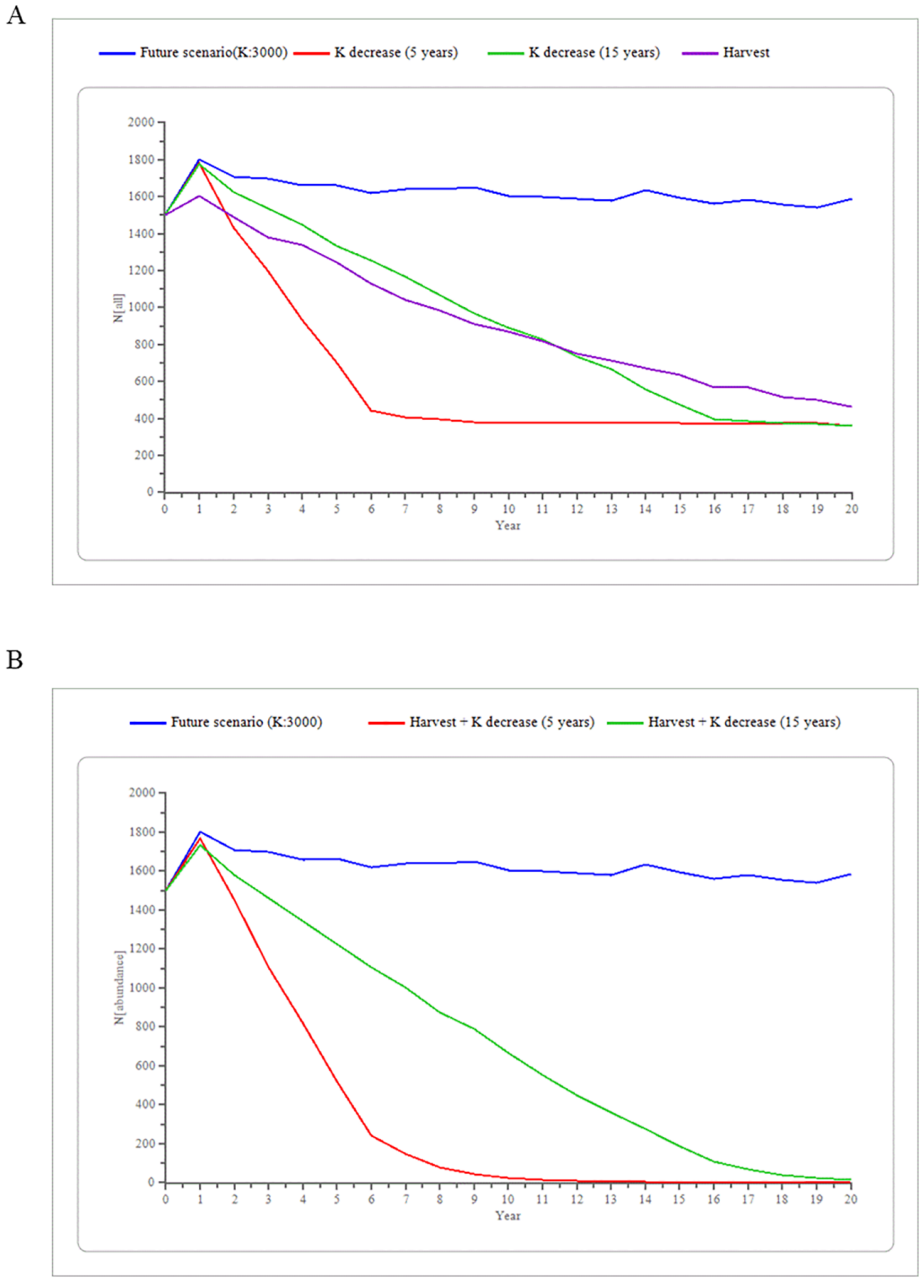
Predicted wild boar population trend under different management strategies. Results of testing the management strategies for the wild boar population of Collserola Natural Park, Spain, in the future scenario show different effectiveness, was a) 80% for the idealistic option (annual decrease of 15% during 5 years), 86% for the realistic option (annual decrease of 5% during 15 years) for the decrease of anthropogenic food resources strategy and 72% for the Selective harvest strategy; and b) 100% for the Combined strategy.

**Fig 6:**
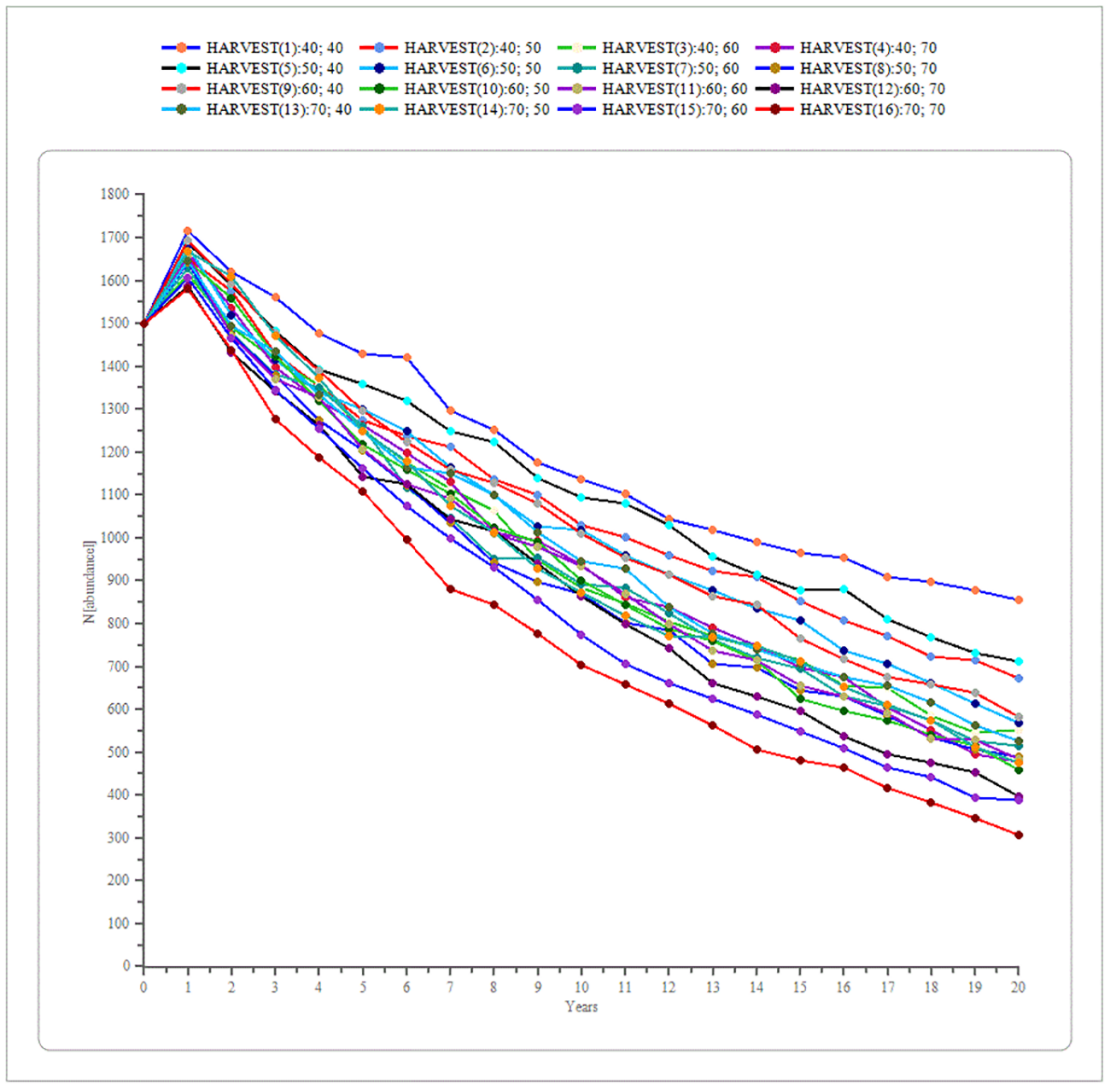
Effects of different harvest values. Sensitivity test outcome showed the relationship between wild boar population size trend and the different harvest values, from 40 to 70 juveniles and yearlings of each sex in the Collserola Natural Park, Spain.

Decreasing the supplementary feeding had an 80% effectiveness to reach the target population value (fixed at 500 wild boar), with a 20% probability of decreasing the population a 58.6% (621 wild boar remaining) at the end of the modeled period for the idealistic decreasing rate option. The conservative decreasing rate option had an 86% effectiveness to reach the target population value, with a 14% probability of achieving a population decrease of 59.1% (614 wild boar remaining) at the end of the modeled period (Table 4, Fig 5A). The sensitivity test in the harvest value of juveniles and yearlings selected 240 individuals, 60 from each sex within each age category, as the most efficient and effective value for the selective harvest strategy (Fig 6). This strategy had 72% effectiveness of reaching the target population value, but also a probability of 28% of a 7.4% increase in population size (1,611 wild boar) at the end of the modeled period (Fig 5A).

When combining both strategies, the number of harvested wild boar necessary to control the population decreased (200 juveniles and yearling wild boar, 50 from each sex within each age category) while the effectiveness increased, achieving a 100% probability of reducing the population size below 500 wild boar by the end of the study period. The rate of decreasing supplementary feeding determined the time to reach this target population size, five years for the idealistic option and thirteen years for the conservative option (Table 4, Fig 5B).

## Discussion

Our models established the combination of a reduction of supplementary feeding resources and the selective harvest of juveniles and yearlings as the most effective and efficient measures to control the CNP wild boar population and revert the increasing trend. The observed annual increase in wild boar abundance fell within the previous interval reported by [52] (r: 0.211, 0.56 times lower) and [15] (r: 0.742, 1.99 times higher), and our predictive models pointed that under the current conditions and management the CNP wild boar population will continue to increase. Since most of the demographic values used in this study (Table 2) fall within or were obtained from the previously reported intervals for other wild boar populations thriving in Mediterranean environments [36], the results and management applications obtained in our study could serve as a basis for other Mediterranean wild boar populations with supplementary feeding, either urban (e.g. rubbish, direct voluntary feeding, stray cat food) or agricultural (e. g. cereal, corn crops). However, since we used indices of relative abundance (hunting bags) producing high confidence intervals for wild boar population estimations (Table 1), our models would gain accuracy by using more reliable wild boar abundance data, like those obtained through population counts.

The supplementary anthropogenic food resources available to wild boar in the peri-urban and urban areas surrounding and within the CNP has most probably increased CNP carrying capacity above the natural value [7,14]. Summer is the period with highest mortality of wild boar in Mediterranean populations due to the natural scarcity of food and water [43], but supplementary feeding, irrigated green areas and artificial fountains provide food, water and thermoregulation for wild boar, thus avoiding the natural constraints of foraging on demographic effect [52]. In the CNP population, the incidences in urban areas are mostly caused by juveniles and females with piglets in good nutritional conditions in summer [28], suggesting that the availability of anthropogenic resources in (peri-)urban areas compensate the aforementioned natural environmental constraints. This artificial food supply and the consequent reduction in mortality makes difficult the estimation of the real carrying capacity of CNP by direct methods.

Mediterranean wild boar populations consist predominantly of juveniles and yearlings, with high reproductive rates and high mortality in the first year of life [1]. Wild boar have one of the highest fecundity rate among ungulates under good conditions [53] and can even increase under strong hunting pressure, because of increased reproductive output of yearling females, which are recruited sooner and in a greater percentage [18,37]. Therefore, Mediterranean wild boar populations are characterized by intense responses to food availability and weather conditions, resulting in sudden increases in numbers [39,44]. Under such conditions, generation time may be as low as two years, a value typically observed for rodents or passerine birds [18]. Population dynamics of wild boar population under favorable conditions seem rather typical for *r*, fast-life strategists or at an intermediate position along the capital–income continuum than for medium-sized ungulates [9,38, 49, 51,53,54]. Altogether, the supplementary food available and the capability of wild boar of exploiting these resources explain the increasing trend observed in the CNP wild boar population, and consequently the relevance of reducing such food resources to revert this trend.

The current hunting management strategy has not achieved a reduction in the CNP wild boar population increase, but maintains the CNP wild boar population approximately half (i.e. 1,500 individuals) of the HPT value (i.e. 3,000 individuals). Traditional battues focus on adult wild boar whose mortality has little if any impact on the demography of the CNP wild boar population, whereas our models point juveniles and yearlings as the age classes to target in order to achieve a significant reduction in the CNP wild boar population. The effect of yearling male mortality was low, but distinguishing male and female juvenile and yearling wild boar is rarely feasible when hunting Mediterranean bush environments. These results agree with previously reported results in other wild boar populations, where the sensitivity of juveniles and yearlings were higher under good environmental conditions [18,38,50], but are opposite to others where adult survival had the highest sensitivity in a growing population [15]. This higher effect of juvenile and yearling mortality on population dynamics is likely related to the increased offspring production, piglet survival and population recruitment due to the overabundance of anthropogenic resources in CNP and the AMB.

Decreasing the percentage of breeding females did not seem a feasible target for reducing the CNP wild boar population, since it would be necessary to restrain the percentage of adult breeding females below 30% in order to appreciate significant effects on the population size. Future approaches to fertility control achieved through feeding may be able to target a much higher proportion of the population for a given effort, thus making fertility control a feasible option in restricted areas such as urban or protected areas (i.e. National parks).

When assessing the most efficient and effective measures to reduce the CNP wild boar population selected by the models, decreasing CNP supplementary anthropogenic resources modified both HPT (K value in VORTEX model) and mortality rates for all age classes (being therefore less specific). On the other hand, selective harvest had a strong effect on the mortality rate of specific age classes. Considering each management strategy separately, decreasing supplementary anthropogenic food resources has the strongest total effect, whereas selective harvesting is more effective and easier to implement, although reducing juvenile and yearling population might be more challenging than reducing adult wild boar population. However, the combination of both strategies reached 100% of effectiveness in achieving the management objectives (Table 3) and decreased the number of harvested wild boar required to control the population growth.

The agreement between the modeled and the estimated wild boar population trend from 2000 to 2015 indicated that the model was at least one of the possible explanations and that the carrying capacity, mortality and breeding rates used were reasonable. Our study showed the utility of PVA models as a species control management tool, for indirectly determining carrying capacity through the analysis of past scenarios, predicting population trend, and testing and targeting the sensitivity of biological variables and management strategies. This allows to design efficient and effective management plans prior to undertaking any action, increasing the effectiveness of management efforts through saving money and resources under the usually limited budgets. Though the final evaluation of the application of the results will require budgeting of the management actions, this was beyond the objective of the present study. That cost depends on the area and hence budget must be individually quantified for every particular context and management. PVA has also limitations, such as being usually focused on a single species, needing more data than other methods and, in many circumstances, the wide confidence limits of the estimates of extinction time produce meaningless results, unless used to compare the relative values of different management strategies [21,22].

## Conclusions

The combination of decreasing carrying capacity by reducing supplementary food and focusing the harvest effort on the demographically most relevant age categories (i.e., juveniles and yearlings) revealed as the most efficient management strategy to control an increasing wild boar population in a Mediterranean periurban environment with supplementary food over the natural resources. These strategies will probably be also the most efficient ones in other over supplemented increasing wild boar populations in similar situations, although studies should be carried out in a case by case basis in order to fine-tune the best management option and their specific efficacy and efficiency in each context.

Decreasing supplementary feeding involves natural, environmental and social factors. Management efforts should focus on (1) voluntary feeding control; (2) stray cat food; (3) waste collection; and (4) management of green areas in the CNP and its surroundings, including urban green spaces. Increasing night waits under special permits would allow targeting the vulnerable life stages, since they are more selective and efficient than traditional battues [55,56]. The capture of juveniles and yearlings wild boar using specially designed traps could also be an alternative option.

Our PVA allowed the prediction of the future trend of the CNP wild boar population under the current environmental conditions and management, validated by the agreement with the population trend observed in the past. Moreover, PVA also assessed the efficacy and efficiency of potential management strategies previously to their implementation, saving efforts and money by identifying those with more potential impact on the CNP wild boar population. This approach can be useful in other populations and scenarios not only for wild boar, but as a previous step before implementing management measures also for any other species.

## Supporting information

S1 Table Vortex functions. Harvest and breeding functions used in the model of the Collserola Natural Park wild boar population.
(PDF)

S2 Table Hunting data. Wild boar captured, hunted or found dead from 2000 to 2015, used to calculate population and specific sex and age classes relative abundances and mortality rates.
(XLSX)

S1 Fig Urban wild boar. Wild boar population in the study area habituated to humans showing A) indirect and B) direct feeding from anthropogenic resources.
(PDF)

S2 Fig Relationship between Hypothetical Population Threshold (HPT) and wild boar population trend. Sensitivity test outcome of the Hypothetical Population Threshold (HPT): Supplementary feeding availability (K value in VORTEX model), showed a total variation in the Collserola Natural Park wild boar population size of 3,820 individuals and an effect of 9.96%. Each line represents the population projection for the different HPT values (from 500 to 6500, increasing by 500).
(PDF)

S3 Fig Relationship between mortality and wild boar population trend. Sensitivity test outcome for the mortality rates of the wild boar population of Collserola Natural park (showed different values in variation in population size and effect for Juvenile a) males and b) females (2,000; 5.75%), Yearling c) males and d) females, and Adult e) males and f) females. Each line represents the population projection for the different mortality values (from 0% to 100%, increasing by 10%).
(PDF)

S4 Fig Relationship between fertility and wild boar population trend. Sensitivity test outcome for the fertility rates showed different values in variation in the Collserola Natural Park wild boar population size and effect for a) males and b) females, c) Juvenile females, d) Yearling females, and e) Adult females. Each line represents the population projection for the different breeding values (from 0% to 100%, increasing by 10%).
(PDF)
