# Supplementary material for: Stochastic assessment of management strategies for a Mediterranean peri-urban wild boar population
Source: PLoS One. 2018 Aug 29;13(8):e0202289. doi: 10.1371/journal.pone.0202289 (PMC6114779; doi:10.1371/journal.pone.0202289)
Supplement: S1 Table — Harvest and breeding functions used in the model of the Collserola Natural Park wild boar population. (PDF) [file pone.0202289.s001.pdf]

MODEL PARAMETER	FUNCTION
**HarvestPastScenario**	=((Y<5)*((A<2)*((8,6*((10*N)/100)/100))+((A=2)+(A=3)+(A=4)+(A=5)+(A=6)+(A=7))*((5,4*((10*N)/100)/100))))+ ((Y=5)+(Y=7)+(Y=8)+(Y=10))*((A<2)*((8,6*((15*N)/100)/100))+((A=2)+(A=3)+(A=4)+(A=5)+(A=6)+(A=7))*((5,4*((15*N)/100)/100)))+ ((Y=11)*((A<2)*((8,6*((20*N)/100)/100))+((A=2)+(A=3)+(A=4)+(A=5)+(A=6)+(A=7))*((5,4*((20*N)/100)/100))))+ ((Y=12)*((A<2)*((8,6*((25*N)/100)/100))+((A=2)+(A=3)+(A=4)+(A=5)+(A=6)+(A=7))*((5,4*((25*N)/100)/100))))+ ((Y=13)*((A<2)*((8,6*((30*N)/100)/100))+((A=2)+(A=3)+(A=4)+(A=5)+(A=6)+(A=7))*((5,4*((30*N)/100)/100))))+ ((Y=14)*((A<2)*((8,6*((50*N)/100)/100))+((A=2)+(A=3)+(A=4)+(A=5)+(A=6)+(A=7))*((5,4*((50*N)/100)/100))))
**Harvest Future Scenario**	=((A<2)*((8,6*((30*N)/100)/100))+((A=2)+(A=3)+(A=4)+(A=5)+(A=6)+(A=7))*((5,4*((30*N)/100)/100))
**Female Reproduction**	=(15*(A=0))+(60*(A=1))+(70*(A>1))
