# Supplementary figures and images for: Stochastic assessment of management strategies for a Mediterranean peri-urban wild boar population
Source: PLoS One. 2018 Aug 29;13(8):e0202289. doi: 10.1371/journal.pone.0202289 (PMC6114779; doi:10.1371/journal.pone.0202289)

A

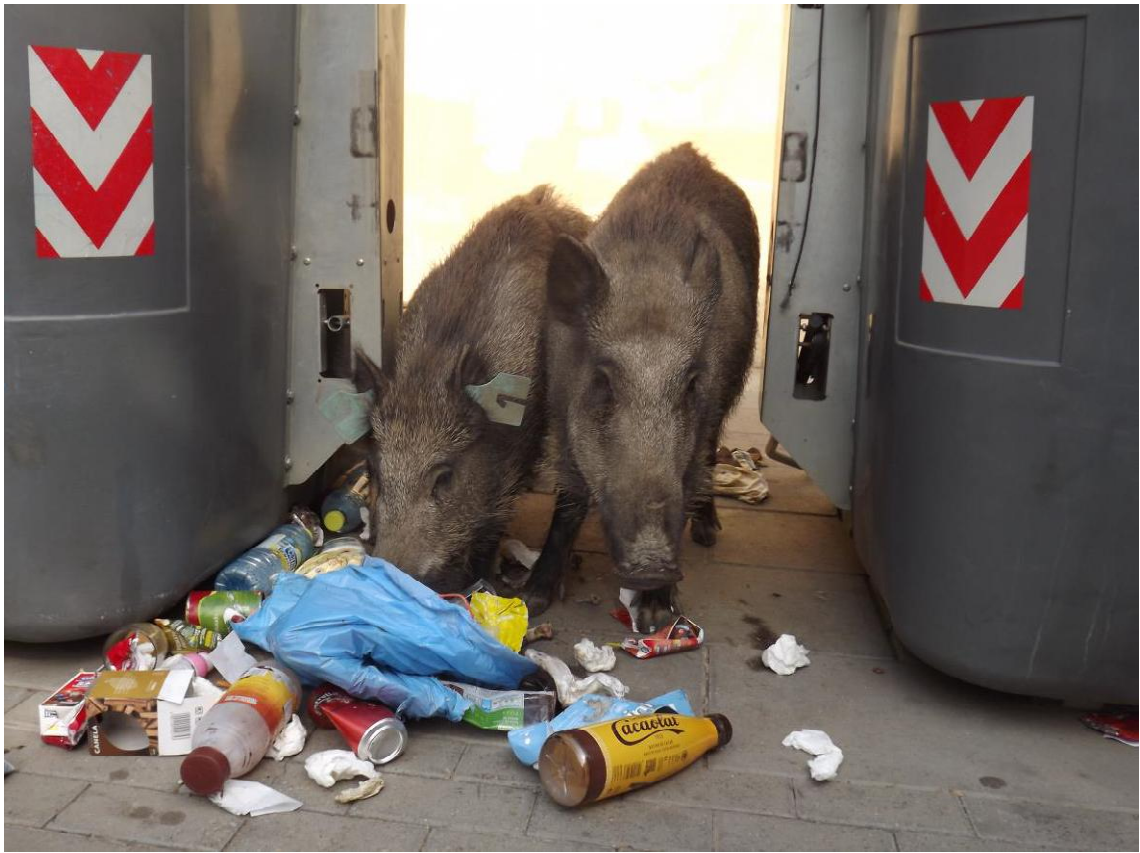

B

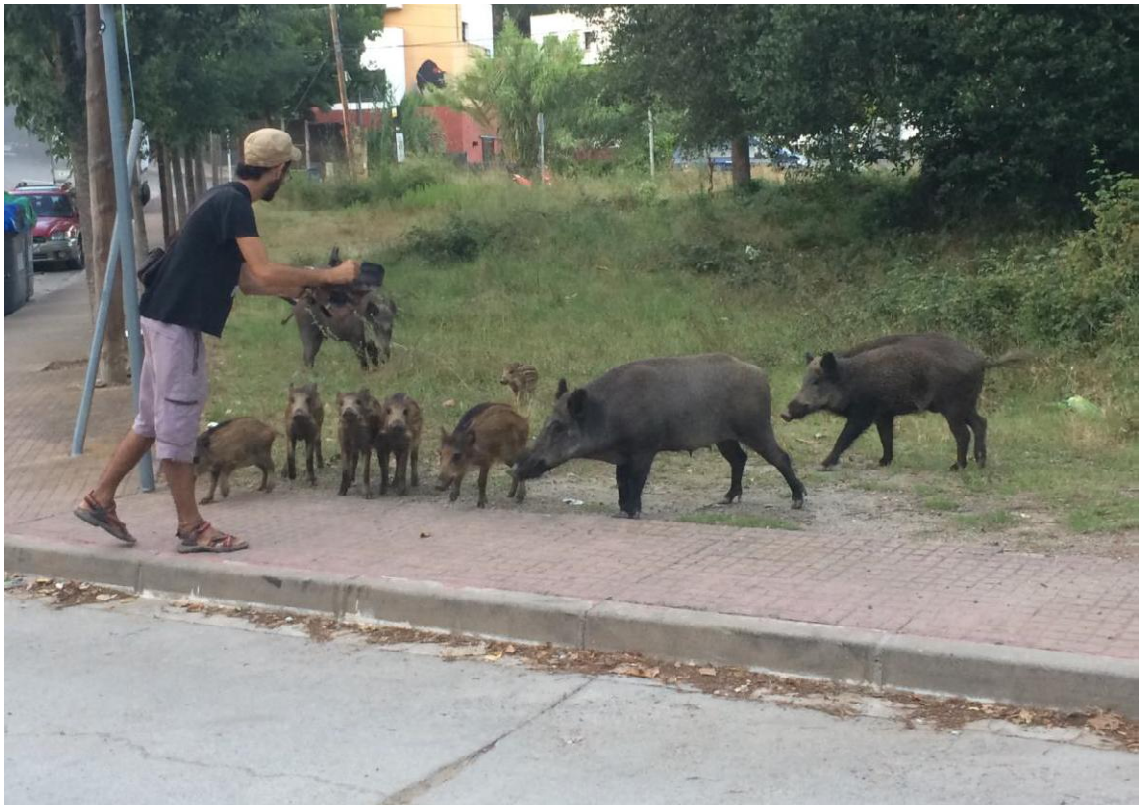

Supplement: S1 Fig — Wild boar population in the study area habituated to humans showing A) indirect and B) direct feeding from anthropogenic resources. (PDF) [file pone.0202289.s003.pdf]

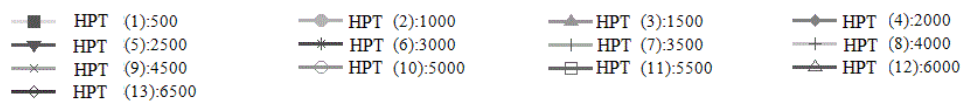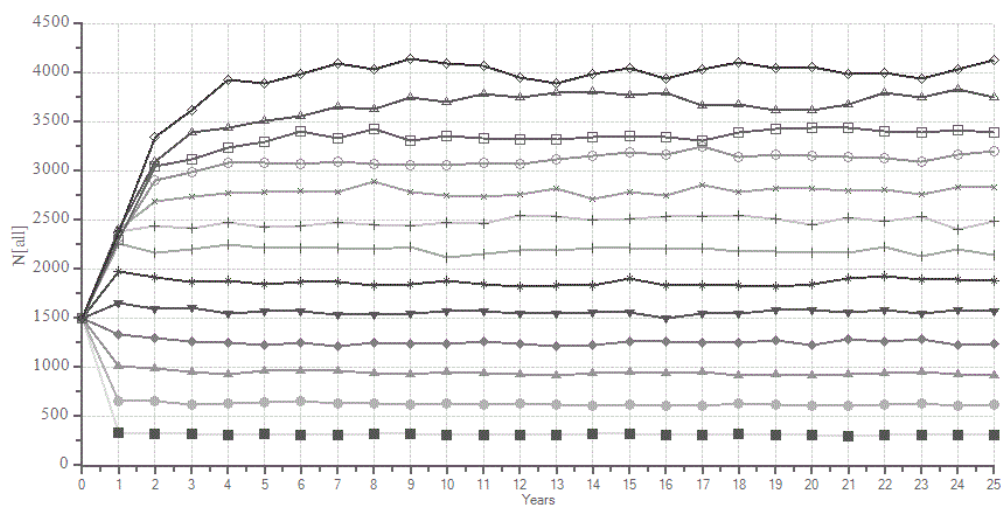

Supplement: S2 Fig — Sensitivity test outcome of the Hypothetical Population Threshold (HPT): Supplementary feeding availability (K value in VORTEX model), showed a total variation in the Collserola Natural Park wild boar population size of 3,820 individuals and an effect of 9.96%. Each line represents the population projection for the different HPT values (from 500 to 6500, increasing by 500). (PDF) [file pone.0202289.s004.pdf]

A

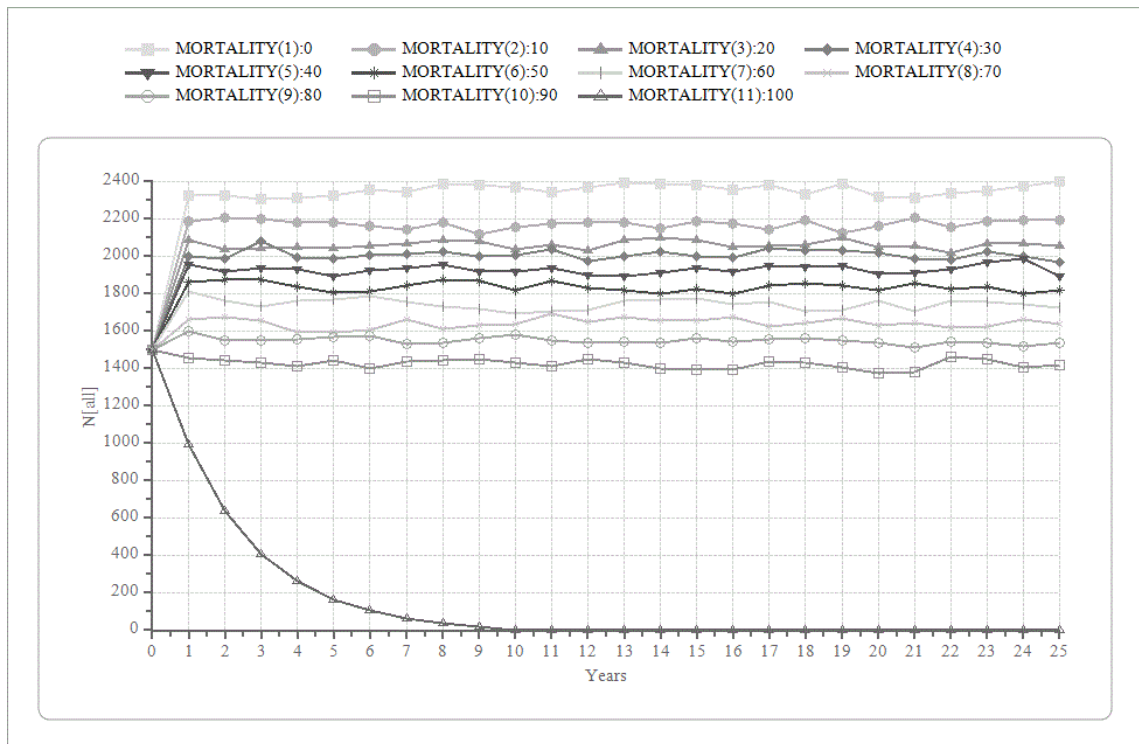

B

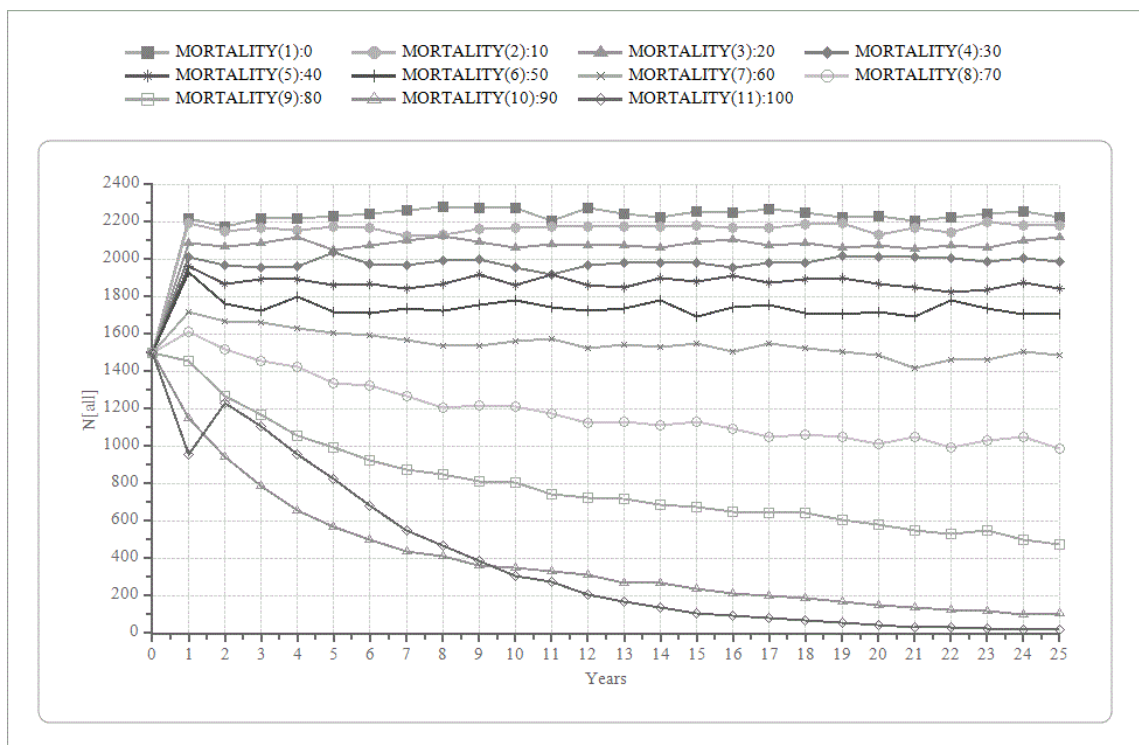

C

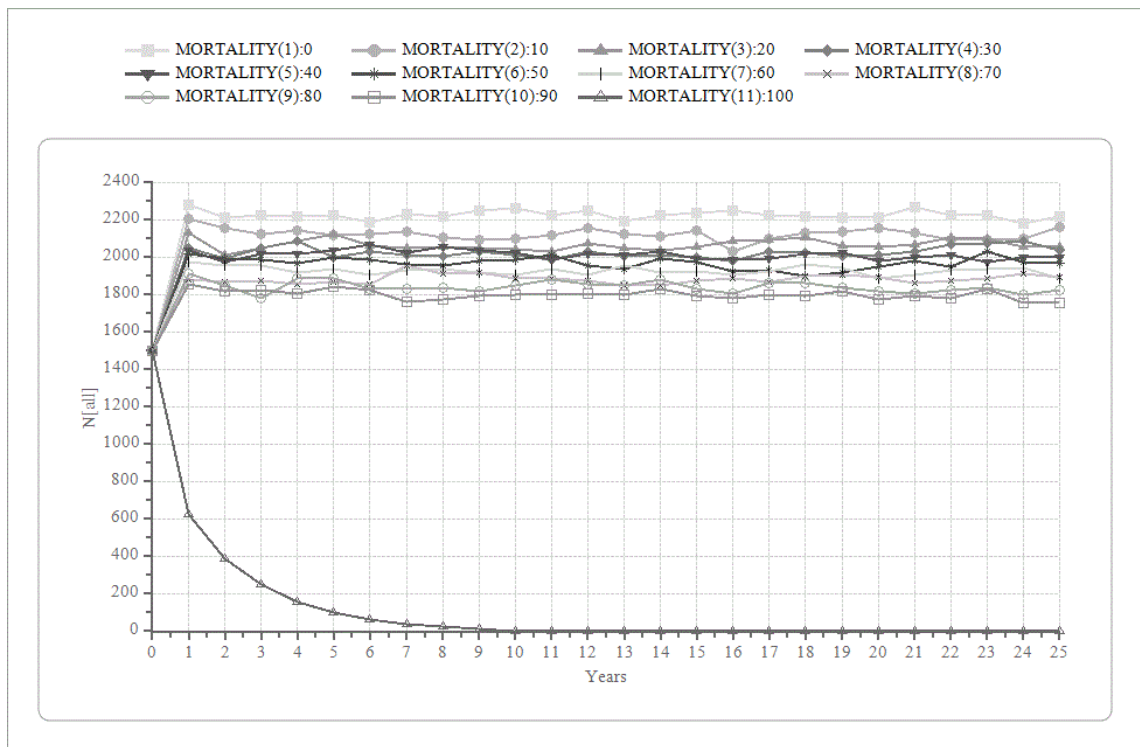

D

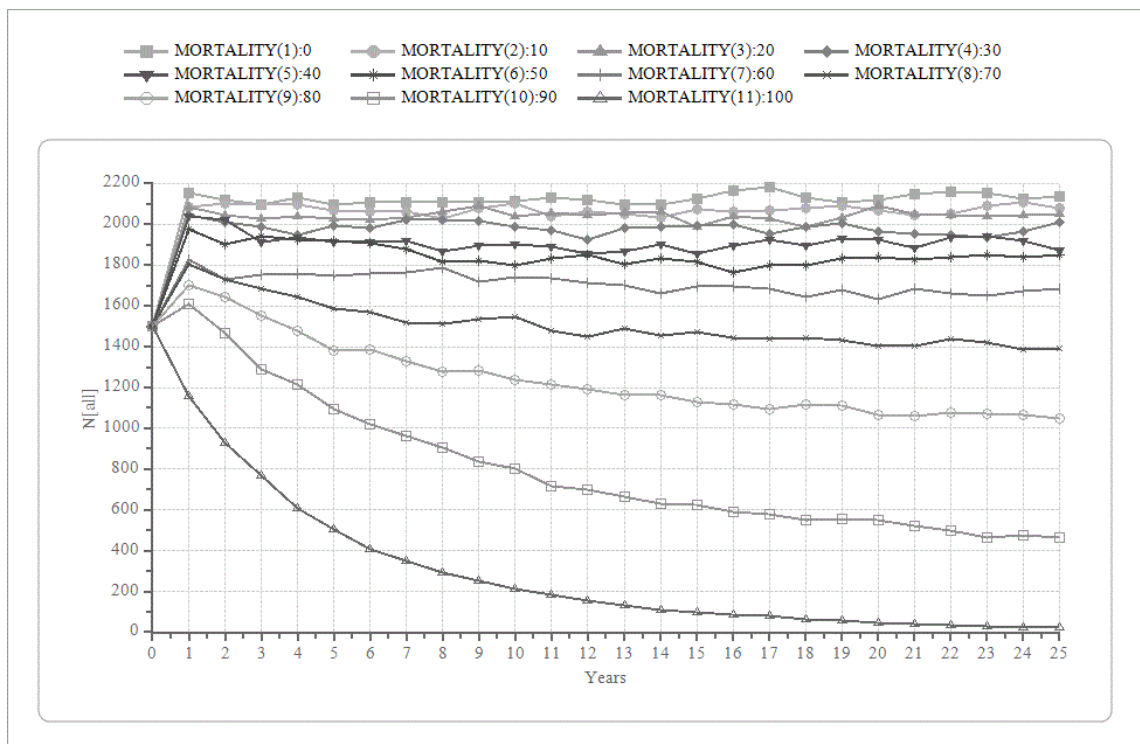

E

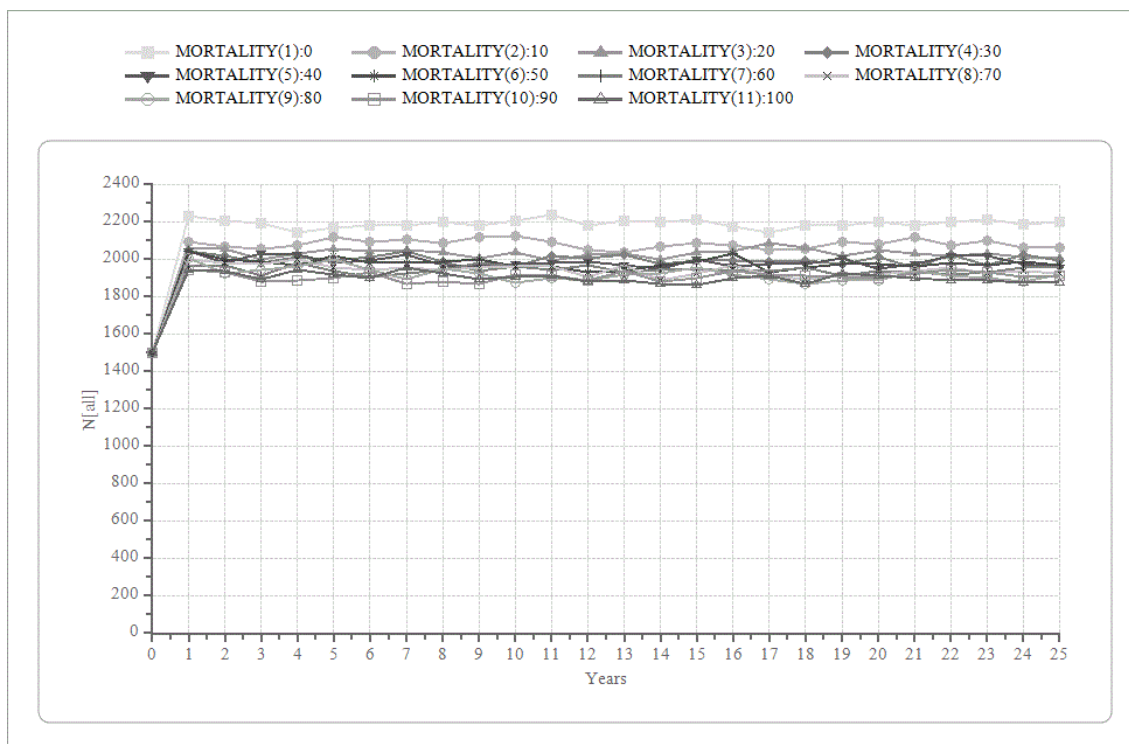

F

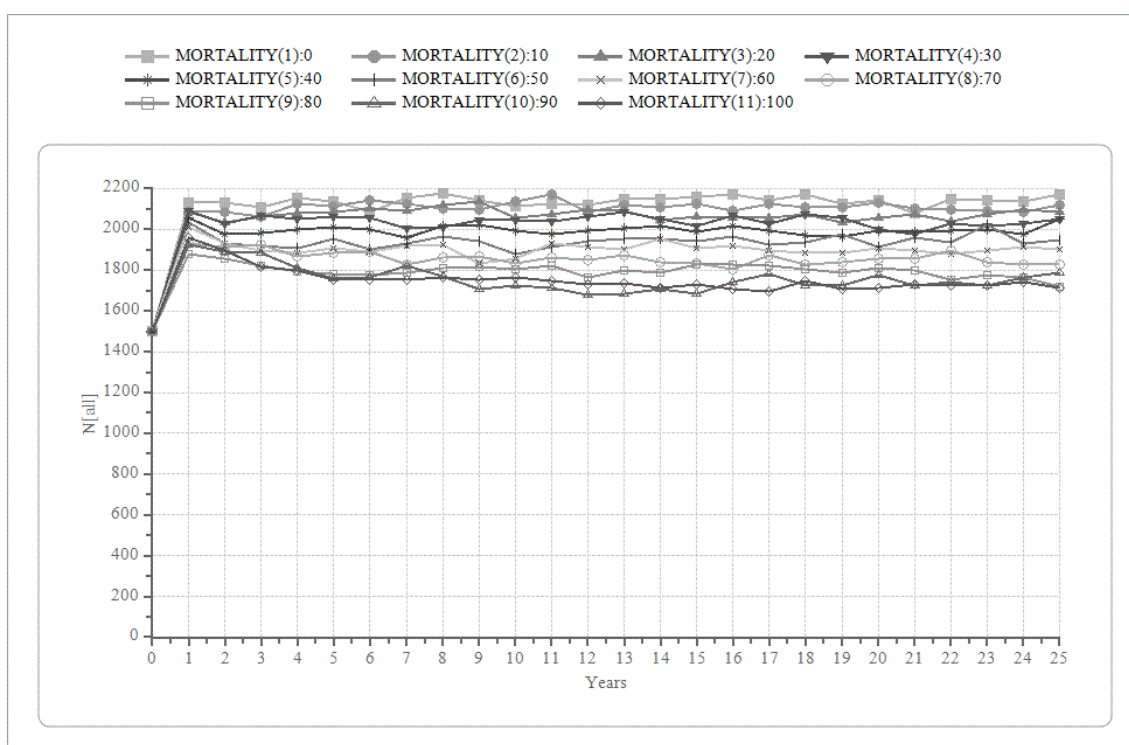

Supplement: S3 Fig — Sensitivity test outcome for the mortality rates of the wild boar population of Collserola Natural park (showed different values in variation in population size and effect for Juvenile a) males and b) females (2,000; 5.75%), Yearling c) males and d) females, and Adult e) males and f) females. Each line represents the population projection for the different mortality values (from 0% to 100%, increasing by 10%). (PDF) [file pone.0202289.s005.pdf]

A

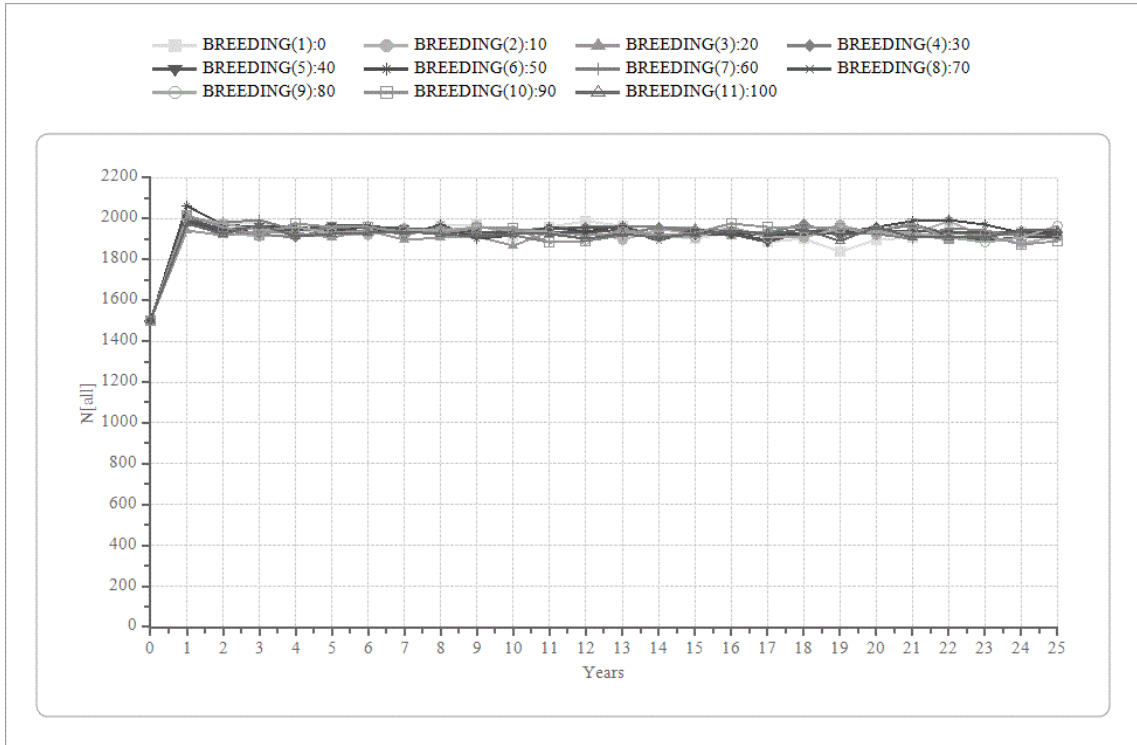

B

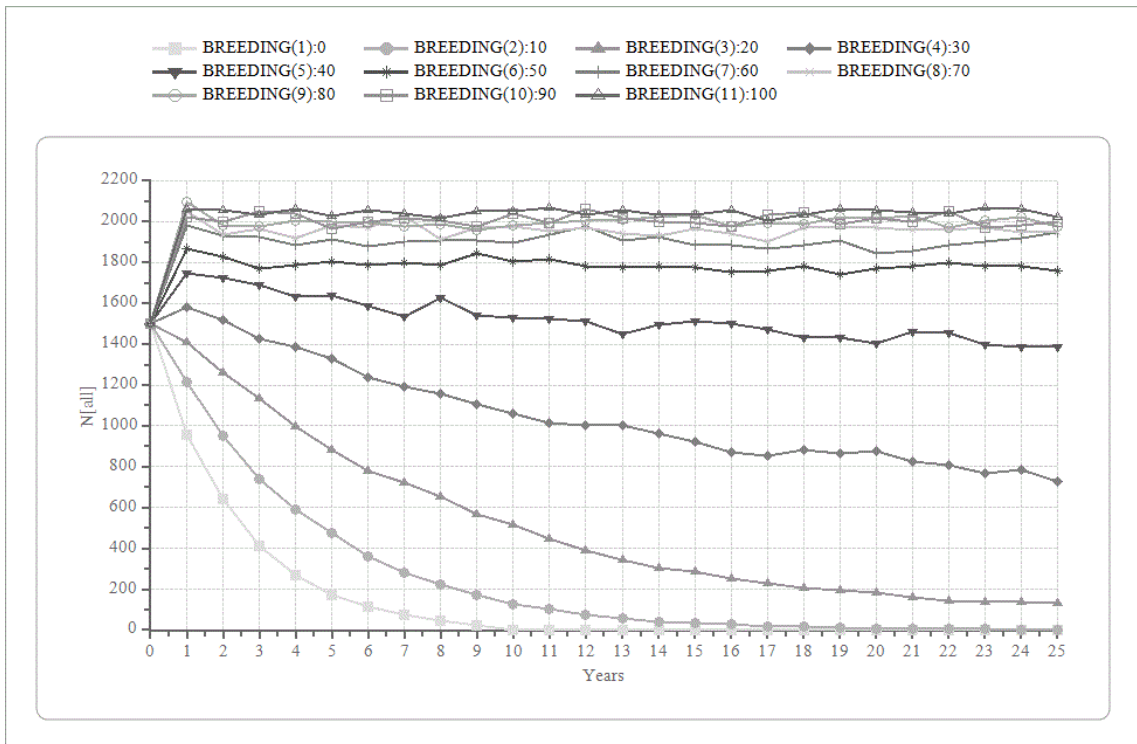

C

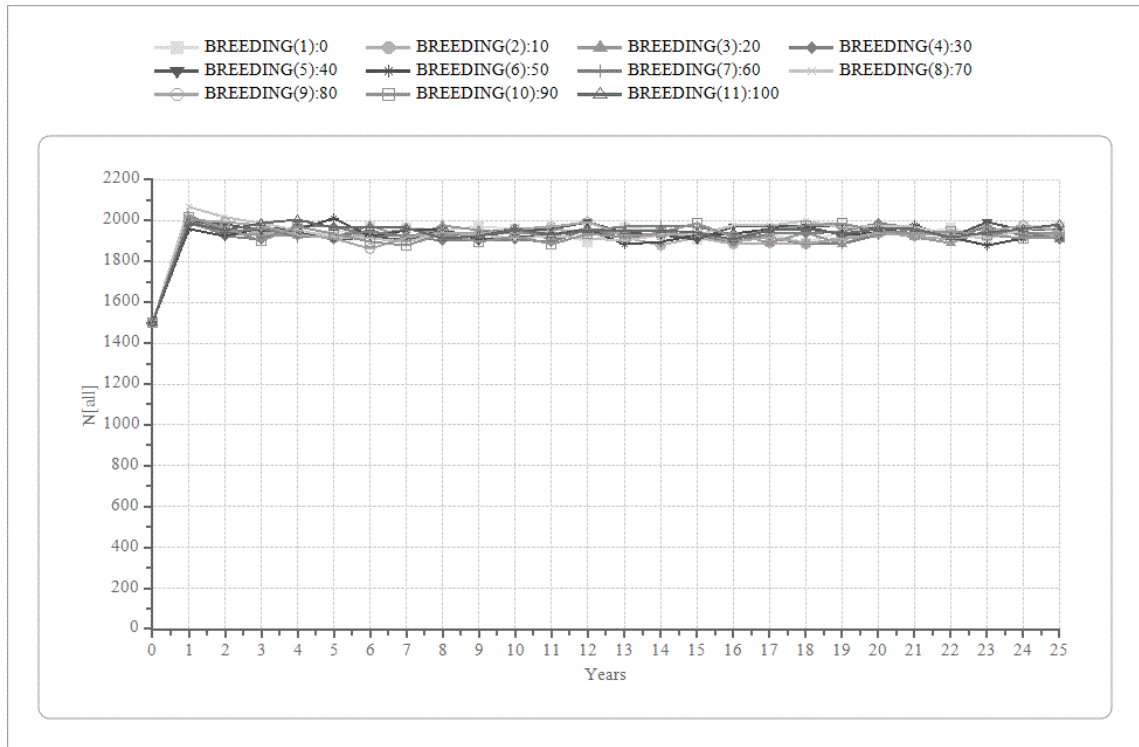

D

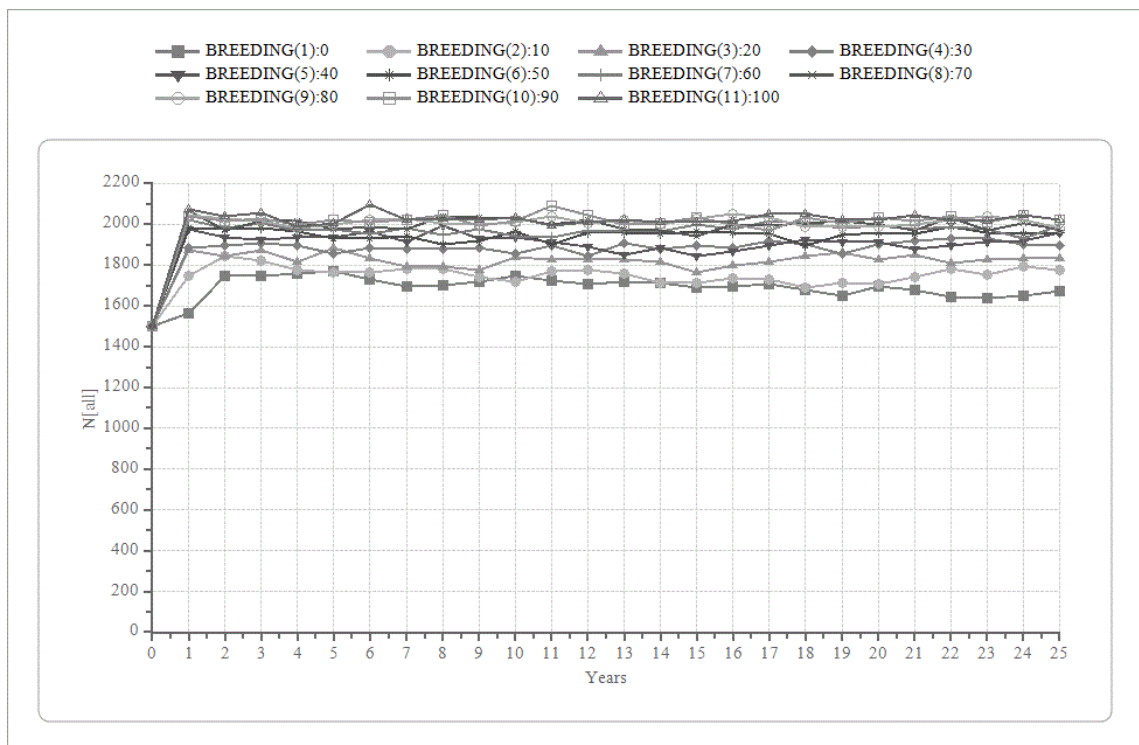

E

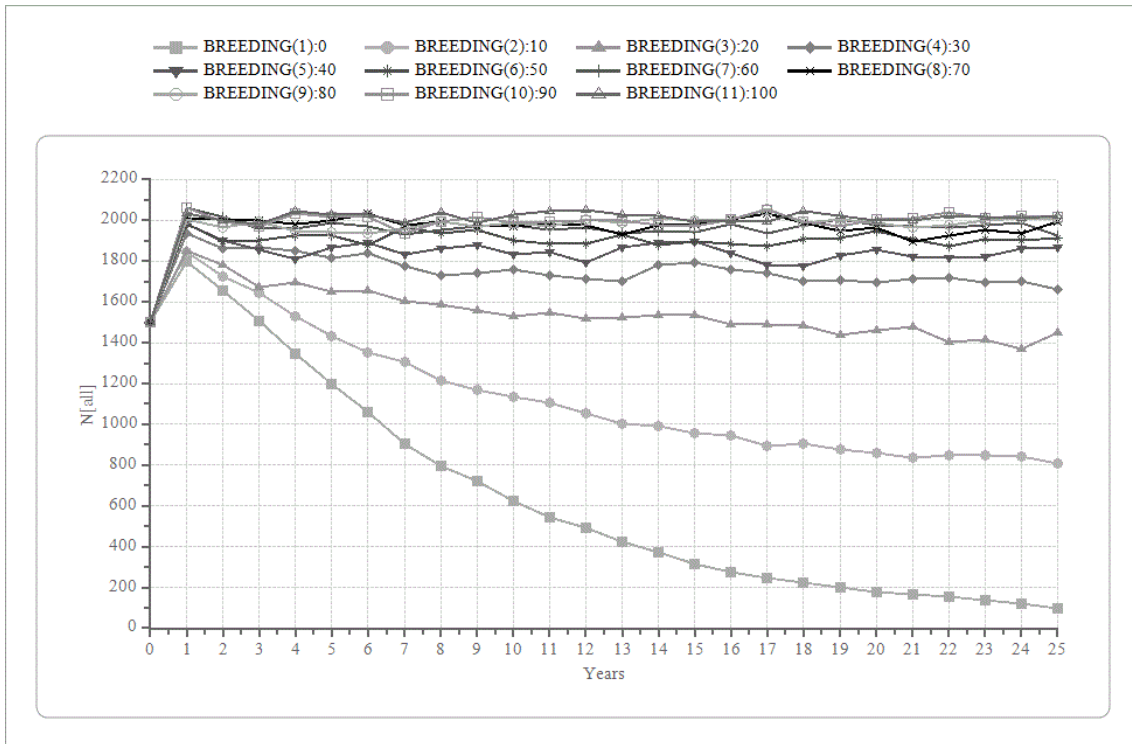

Supplement: S4 Fig — Sensitivity test outcome for the fertility rates showed different values in variation in the Collserola Natural Park wild boar population size and effect for a) males and b) females, c) Juvenile females, d) Yearling females, and e) Adult females. Each line represents the population projection for the different breeding values (from 0% to 100%, increasing by 10%). (PDF) [file pone.0202289.s006.pdf]
